# Histologic and Histomorphometric Analysis of the Effects of Bisphosphonate and Parathyroid Hormone with Bone Graft on Bone Healing and Formation in Rabbit Models

**DOI:** 10.34172/joddd.41940

**Published:** 2026-03-30

**Authors:** Omid Soltaninia, Keyhan Soleimani, Fatemeh Mashhadi Abbas, Mohammadreza Khoshtarigh

**Affiliations:** ^1^Department of Oral and Maxillofacial Surgery, School of Dentistry, Hamadan University of Medical Sciences, Hamadan, Iran; ^2^Department of Pathology, Shahid Beheshti University of Medical Sciences, Tehran, Iran; ^3^Oral and Maxillofacial Surgery Department, School of Dentistry, Tehran University of Medical Sciences, Tehran, Iran

**Keywords:** Anima model, Bisphosphonates, Bone defects, Bone grafting, Bone regeneration, Maxillofacial development, Teriparatide

## Abstract

**Introduction:**

The standard method to accelerate healing of large bone defects involves using bone grafts alone or with biomaterials. Given the effects of bisphosphonates and parathyroid hormone (PTH) on bone remodeling, and the limited and contradictory studies available, this study aimed to evaluate the histologic and histomorphometric effects of combining bisphosphonate (zoledronic acid) and PTH (teriparatide) with bone grafts on bone healing and formation in rabbit models.

**Methods:**

Bilateral defects were created in the tibias 35 New Zealand white rabbits, divided into four groups, including negative control (no treatment) (n=5), positive control (bone powder) (n=10), intervention 1 (zoledronic acid+bone powder) (n=10), and intervention 2 (teriparatide+bone powder) (n=10). In each of the groups, half of the rabbits were euthanized in the 8th week and the other half in the 16th week after surgery, and their tibia bones were removed for histologic and histomorphometric evaluations. The outcomes were new bone formation, remaining bone graft percentage, foreign body reaction, and inflammation levels.

**Results:**

The highest percentage of new bone formation in the 8th week was observed in the intervention 2 group; in the 16th week, it was observed in the negative control group. However, the difference between the groups was not significant neither in the 8th week nor in the 16th week (*P*>0.05). The percentage of remaining bone graft in the intervention 1 group in the 8th and 16th week was significantly higher than in other groups (*P*<0.05). In week 8, the amount of inflammation in the intervention 1 group was non-significantly higher than in the intervention 2 group (*P*>0.05), and in week 16, the amount of inflammation in the intervention group 1 was significantly higher than in the other two groups (*P*<0.05). The highest frequency of foreign body reaction in the 8th and 16th weeks was related to the intervention 1 group (*P*<0.05).

**Conclusion:**

Overall, teriparatide showed favorable outcomes. Teriparatide and bisphosphonate exhibited distinct and potentially complementary effects. Teriparatide promoted new bone formation and resorption, while bisphosphonates reduced bone graft resorption and caused inflammation and foreign body reactions. Further large-scale animal studies on teriparatide and bisphosphonate are recommended. These findings suggest time-dependent effects, with teriparatide potentially accelerating early healing and bisphosphonates prolonging graft persistence, though larger studies are necessary to confirm non-significant trends.

## Introduction

 Defects of large bones, which are mostly due to accidents or trauma, still pose management challenges.^1^ Bone lesions in the craniofacial region often present with significant complications and can arise due to various factors such as trauma, tumors, infections, orofacial clefts, comminuted fractures, bone cysts, congenital defects, and other disorders associated with substantial bone damage.^2^ Medical intervention for repair is essential in these cases.^3^ Moreover, the fractures can negatively impact the quality of life, occupation, physical, and mental health, with high economic burden.^4^ Therefore, several strategies have been developed to accelerate the healing time and reduce the adverse effects of these defects. In this regard, bone grafts are frequently employed to recover lost bone in a highly acceptable, technical, and skillful way that allows for the restoration of the form and function of bones.^5^ Bone graft should be osteoconductive, osteoinductive, and osteogenic while minimizing patient risks; they must also be affordable, reduce infection risk, ensure biocompatibility, and have structural similarity to bone in terms of porosity and mechanical strength.^6^

 In recent years, bone resorption inhibitors and anabolic agents have been developed as bone remodeling treatments. Bone remodeling is a continuous process of bone matrix mineralization, destruction, and formation, which is regulated by three key factors: parathyroid hormone (PTH), vitamin D, and calcitonin.^7^ Anti-resorptive drugs, including bisphosphonates (e.g., etidronate, risedronate, alendronate), raloxifene, denosumab, and odanacatib, influence bone remodeling by inhibiting osteoclast activity and reducing bone resorption.^8^ Bisphosphonates are stable synthetic analogs of inorganic pyrophosphates. They are produced by replacing an oxygen atom in the hydrolyzable phosphorus-oxygen-phosphorus (P-O-P) structure of the pyrophosphate molecule with a carbon atom. This substitution results in the formation of a hydrolysis-resistant carbon-phosphorus (C-P) bond. These compounds are used in the treatment of conditions such as osteoporosis and other musculoskeletal disorders and bone diseases.^9^ They also manage bone and joint conditions such as bone metastases, fibrous dysplasia, Charcot arthropathy, Paget’s disease, and developmental disorders.^10^

 The PTH is an 84-amino acid protein and a key calcium homeostasis factor in the body, playing a crucial role in bone metabolism.^11^ PTH has both direct and indirect effects on bone remodeling, maintains optimal calcium concentrations, and plays a role in bone turnover, renal phosphate excretion, the activation of vitamin D, and the treatment of osteoporosis.^12^ The continuous and excessive production of PTH has catabolic effects on bone, while it has been shown that pulse therapy with PTH increases the number, maturation, and activity of new osteoblasts and reduces osteoblast apoptosis.^13^

 Considering the role of PTH in enhancing bone formation and its clinical application in treating bone diseases, its use in conjunction with bone graft materials could have beneficial effects.^14,15^ Studies have shown that while bisphosphonates have anabolic activities, they can also exhibit catabolic results like osteonecrosis of jaw.^16^ There are also some discrepancies in animal models regarding the efficacy of PTH or bisphosphonates on bones. Bisphosphonates exhibited some negative impacts on osseointegration in a study.^17^ While another study showed increases in total and new bone volumes in PTH in rabbits models.^18^ Some studies have explored the combination of bisphosphonates and PTH, demonstrating enhanced bone architecture and strength in animal models,^19,20^ while others have noted limited additive effects.^21^ Therefore, the present study aimed to histologically and histomorphometrically evaluate the effects of combining bone grafts with PTH and bisphosphonates separately on bone formation and repair in rabbit models. The following null hypothesis was tested in this study: There would be no significant difference in the histologic and histomorphometric outcomes (new bone formation, remaining bone graft percentage, foreign body reaction, and inflammation levels) between the groups treated with bisphosphonate (zoledronic acid) or PTH (teriparatide) combined with bone grafts and the control groups (bone grafts alone or no treatment) in rabbit tibial defect models.

## Methods

###  Experimental Models

 This study was conducted from 2019 to 2021 at Hamadan University of Medical Sciences, Hamadan, Iran. The research population consisted of 35 healthy New Zealand white rabbits aged at least 6 months. The rabbits were sourced from a certified breeding facility, weighing between 2.5 and 3.5 kg. Inclusion criteria required rabbits to be free of any visible signs of disease, with normal appetite, mobility, and behavior as assessed by a veterinarian prior to enrollment. Exclusion criteria included rabbits showing signs of illness (e.g., weight loss, lethargy, or respiratory issues), infection, or abnormal behavior before or during the study, as well as those lost to follow-up due to unexpected mortality or surgical complications. Rabbits that became ill or were lost before the completion of interventions were excluded from the study.

 Following the creation of bone defects, the rabbits were categorized as follows: group 1, the negative control, receiving no treatment for the induced bone defect (n = 5); group 2, the positive control, receiving moistened bone powder with saline within the induced bone defect (n = 10); group 3, the intervention group 1, receiving moistened bone powder with bisphosphonate within the induced bone defect (n = 10); and group 4, the intervention group 2, receiving moistened bone powder with teriparatide within the induced bone defect (n = 10). In group 4 (intervention 2), there were 10 rabbits (5 males and 5 females). In the negative control group (group 1), 5 rabbits were included; 2 were sacrificed at 8 weeks (1 male and 1 female), and 3 were sacrificed at 16 weeks (2 males and 1 female).

 The current study was approved by the Ethics Committee of Hamadan University of Medical Sciences, Hamadan, Iran (ethics code: IR.UMSHA.REC.1399.110). This study was carried out in strict accordance with the recommendations in the Guide for the Care and Use of Laboratory Animals of the National Institutes of Health. All efforts were made to minimize suffering.

###  Surgical and Treatment Procedures 

**Figure 1:**
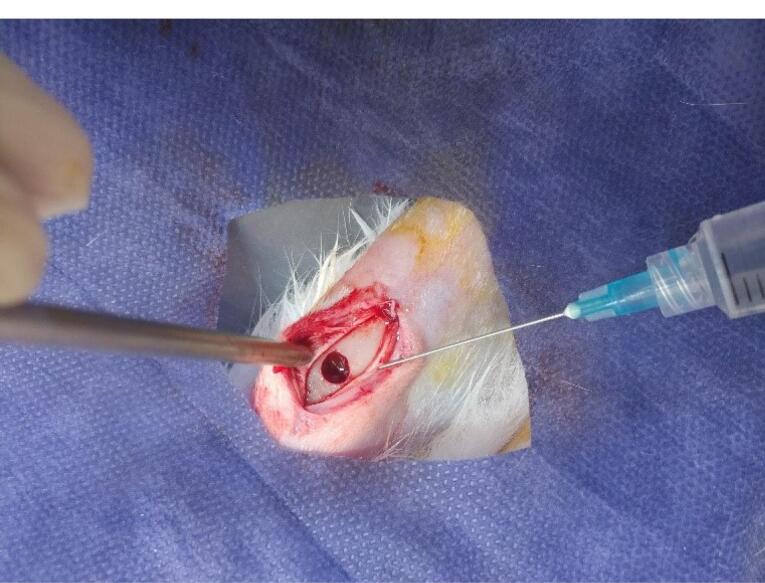
A 2-cm incision on the tibia and the creation of a bone defect

 The rabbits were kept under standard conditions with unrestricted access to food and water. To prevent disuse osteoporosis, the animals were maintained outside of cages for two weeks prior to the surgery. Anesthesia was induced with 6 mg/kg of xylazine and 60 mg/kg of ketamine. To ensure deep anesthesia, they were returned to the cage. Bilateral defects, approximately 5 mm below the knee joint line on the proximal tibia, were selected. The area was prepared by shaving the fur and applying an antiseptic (Betadine) to minimize the risk of infection. Local anesthesia was administered using 2% lidocaine with 1:80000 epinephrine. A 2-cm longitudinal incision was made on the medial aspect of the proximal tibia bone (Figure 1). Following periosteal retraction, a 5-mm-diameter defect was created using continuous irrigation with normal saline delivered through a surgical handpiece, and the resulting defect was irrigated with saline solution.

**Figure 2:**
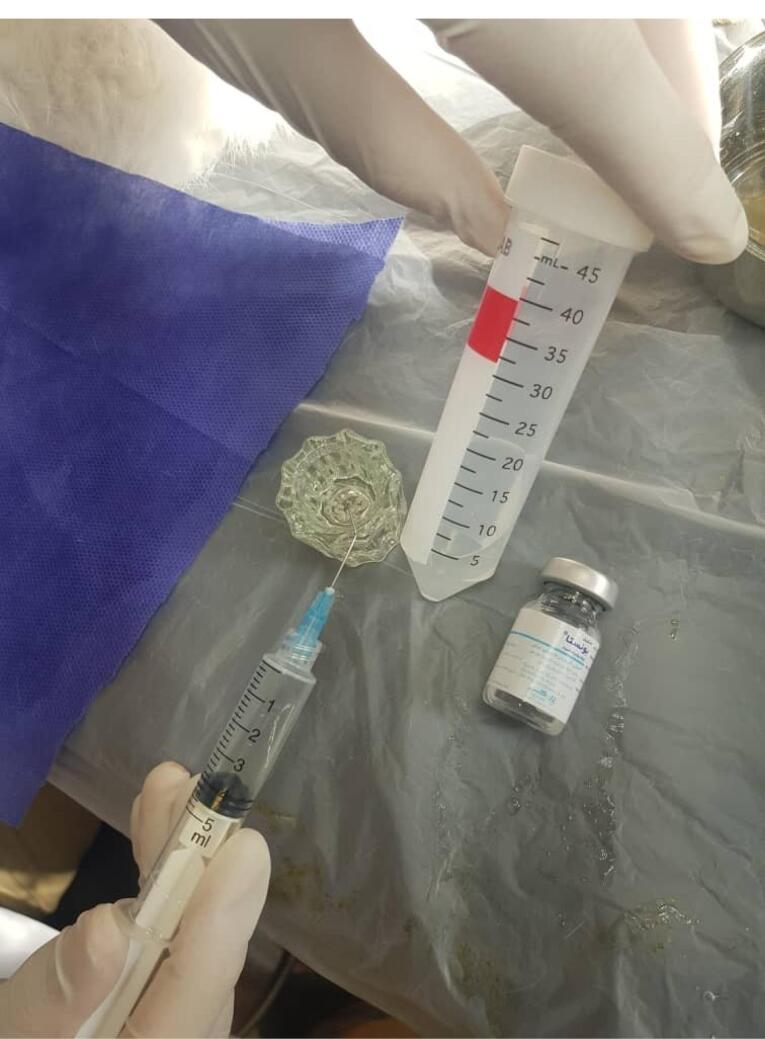
Preparation of diluted zoledronic acid solution

 The amount of bone powder used per bone defect was approximately 0.1 to 0.2 mL, and the type of bone powder was human demineralized bone matrix (DBM) and mineralized bone allograft (MBA) (1000‒2000 µm) (Regen®). Human bone powder was used as a xenograft in rabbits. To prepare the bone powder and bisphosphonate mixture, zoledronic acid (BONSTA® 4 mg/5 mL) at a concentration of 0.005 mg/mL was used. To prepare the 0.005-mg/mL zoledronic acid solution, 0.1 mL of the zoledronic acid solution was diluted with 15.9 mL of distilled water in a laboratory tube (Figure 2). The bone powder was immersed in the diluted zoledronic acid solution for 5 minutes. After this period, the bisphosphonate solution was removed with a syringe. The bone powder was then washed with saline solution by refilling the container with saline, stirring the powder for 30 seconds, and removing the saline with a syringe. This washing process was repeated for 2 minutes.

 To prepare the bone powder and parathyroid hormone mixture, the bone powder was immersed in a container containing 20 µg of teriparatide for 5 minutes. Afterward, the excess teriparatide solution was removed and discarded. In this study, a teriparatide pen (Sinopar) was used. To achieve the required 20 micrograms of teriparatide, the pen dial was turned to number 8, the injection was performed, and the release button was held for 10 seconds to ensure the complete delivery of the specified dose.

 Following surgery, the incision was closed with 4/0 absorbable catgut sutures and the skin was closed with 3/0 silk sutures. To minimize bleeding, pressure was applied to the wound for 5 minutes, followed by the application of tetracycline spray. To reduce pain and prevent infection, flunixin (Flumax M®) was administered as an analgesic and Pen & Strep (Pen & Strep Norbrook®) as an antibiotic, both intramuscularly immediately after the operation. Each rabbit was monitored for three days postoperatively, during which they received a daily dose of flunixin and Pen & Strep intramuscularly.

###  Histomorphometric Analysis

 Regarding sample losses, in the positive control group (group 2), of the five rabbits euthanized using an overdose of ketamine at eight weeks, one was completely lost, and one lost one of its tibias (due to infection). In the same group at 16 weeks, one rabbit was lost. In the intervention group 3 (bisphosphonate), at eight weeks, one rabbit lost one tibia (due to infection). In other groups, half of the rabbits were euthanized at eight weeks postoperatively, and the other half at 16 weeks postoperatively, using an overdose of ketamine. The tibias were then extracted for histomorphometric and histopathologic evaluations. To obtain samples from the defect sites, bone specimens were excised with a margin of at least 5 mm of healthy bone and fixed in 4% formaldehyde for 24 hours. Subsequently, they were decalcified in 10% formic acid for three months. Sections were cut through the diameters, and paraffin blocks were prepared. From these blocks, 5-µm sections with at least three sections per sample were stained using hematoxylin-eosin. These sections were evaluated by a pathologist blinded to the group assignments. Blinding was achieved by assigning random alphanumeric codes to all the samples by an independent technician not involved in the study. The pathologist received only coded slides and was unaware of group assignments until after all evaluations were completed. To ensure standardization, evaluations followed predefined criteria from the American Society for Bone and Mineral Research histomorphometry nomenclature. Intra-observer reliability was assessed by re-evaluating 20% of randomly selected samples (intraclass correlation coefficient > 0.85). Images were captured at × 40 magnification using a Nikon (Eclipse, E400, Japan) digital camera mounted on a Mobin microscope (Ics, Germany).

###  Outcome Evaluation and Data Collection

 The IHMM software (version 1) was used to calculate the percentage of new bone formation relative to tissue volume, the percentage of remaining bone graft relative to the target area, foreign body reaction, and inflammation levels. The software was calibrated using standard reference slides to ensure consistent measurements across samples. The targeted tissue area was identified by the pathologist, and percentage of new bone volume and remaining bone graft area were calculated by the software. The foreign body reaction was determined based on the presence or absence of foreign body giant cells on the histopathological slides. Inflammation was graded from zero to three, with zero indicating no inflammation and three indicating severe inflammation. Grading was standardized using a semi-quantitative scale:

0. no inflammatory cells
1. mild (scattered cells)
2. moderate (focal aggregates)
3. severe (diffuse infiltration)

###  Statistical Analysis

 Data analysis was performed using SPSS 21 (IBM Corp., New York, USA). Descriptive statistics, including frequencies, percentages, means, and standard deviations (SD), were used to summarize the data. Data analysis was conducted using one-way analysis of variance (ANOVA), independent-samples t-test, and Kruskal-Wallis, Mann-Whitney, and chi-squared tests. The normality of the distribution of quantitative variables was assessed using the Shapiro-Wilk test. Potential confounding variables, such as sex and weight, were evaluated for their impact on outcomes using multivariate linear regression where data distribution allowed; no significant confounding effects were found (*P* > 0.05 for all). To minimize bias, group allocation was concealed from the surgical team until intervention, and randomization helped balance baseline characteristics. Due to the small sample size, further sensitivity analyses for bias were not feasible. A P-value of < 0.05 was considered statistically significant.

## Results

**Figure 3:**
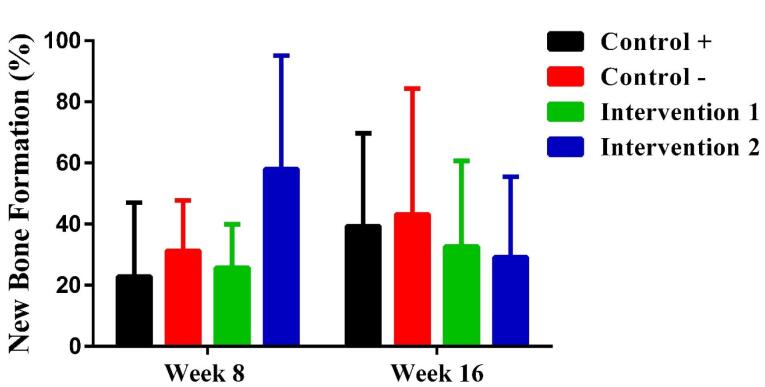
Mean percentages of new bone formation relative to tissue volume in different groups and weeks

 The highest mean percentage of new bone formation relative to tissue volume in the 8th week was observed in the intervention group 2 (58.0 ± 37.1%), followed by the negative control group (31.3 ± 16.4%), the intervention group 1 (25.8 ± 14.1%), and the positive control group (22.9 ± 24.2%). In the 16th week, the highest mean percentage of new bone formation was seen in the negative control group (43.2 ± 41.2%), followed by the positive control group (39.4 ± 30.3%), the intervention group 1 (32.7 ± 28.1%), and the intervention group 2 (29.2 ± 26.3%). There were no significant differences between the groups in the 8th (*P* = 0.112) and 16th weeks (*P* = 0.953). Also, there were no significant differences in each group between 8th and 16th weeks (*P* > 0.05) (Figure 3).

**Figure 4:**
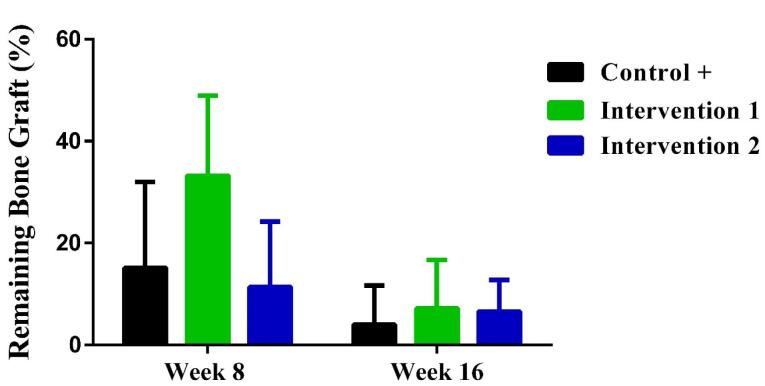
Mean percentages of remaining bone graft relative to the target area in different groups and weeks

 The mean percentage of remaining bone graft relative to the target area in the intervention group 1 was higher than in other groups at both the 8th (33.2 ± 15.7%) and 16th weeks (7.2 ± 9.5%). In the 8th week, the mean percentage of remaining bone graft in the positive control group was higher than in the intervention group 2 (15.2 ± 16.8% vs. 11.4 ± 12.8%), while in the 16th week, the intervention group 2 had a higher mean percentage of remaining bone graft compared to the positive control group (6.2 ± 6.6% vs. 4.0 ± 7.7%). Variations in remaining bone graft were quantified as the percentage of graft material area relative to the total defect area using IHMM software, with means ± SD reported; inter-group differences were assessed via one-way ANOVA or Kruskal-Wallis test, and intra-group temporal changes via paired t-tests or Wilcoxon signed-rank test (post-hoc adjustments applied where significant). There were significant differences between the groups in the 8th (*P* = 0.004) and 16th weeks (*P* = 0.046). Only the intervention group 1 showed significant differences in the remaining bone graft between the 8th and 16th weeks (*P* = 0.001) (Figure 4).

**Figure 5:**
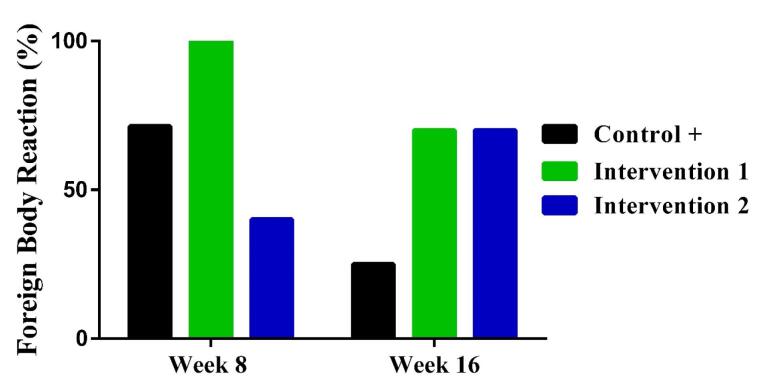
Mean percentages of foreign body reactions in different groups and weeks

 The highest frequency of foreign body reaction in the 8th week was observed in intervention group 1 (100.0%), followed by the positive control group (71.4%), and the intervention group 2 (40.0%). In the 16th week, the highest frequency of foreign body reaction was seen in the intervention groups 1 and 2 (70.0%). In both the 8th (*P* = 0.011) and 16th (*P* = 0.010) weeks, a statistically significant difference in foreign body reaction was observed between the groups. However, the frequency of foreign body reaction in each group in the 8th and 16th weeks was not significantly different (*P* > 0.05) (Figure 5).

**Figure 6:**
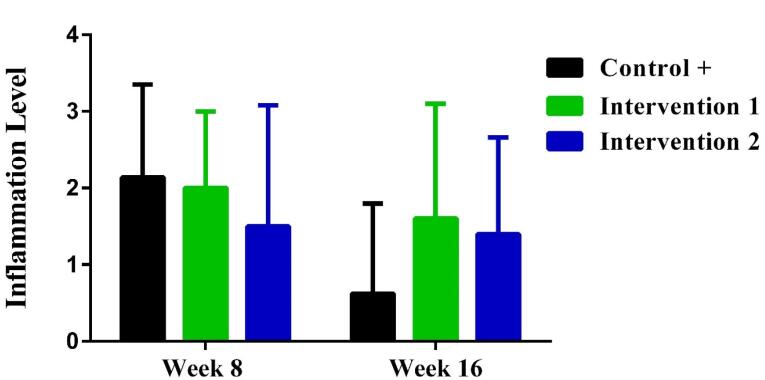
Mean inflammation levels in different groups and weeks

 The highest mean level of inflammation in the 8th week was observed in the positive control group (2.1 ± 1.2), followed by the intervention group 1 (2.0 ± 1.0) and the intervention group 2 (1.5 ± 1.6). In the 16th week, the highest mean level of inflammation was seen in the intervention group 1 (1.6 ± 1.5), followed by the intervention group 2 (1.4 ± 1.3), and the positive control group (0.6 ± 1.2). There were significant differences between the groups in terms of inflammation levels in the 16th week (*P* = 0.039), contrary to the 8th week (*P* = 0.074). Also, there was a significant difference between 8th and 16th weeks in the positive control group (*P* = 0.029) (Figure 6).

## Discussion

 The null hypothesis of this study—there would be no significant difference in the histologic and histomorphometric outcomes (new bone formation, remaining bone graft percentage, foreign body reaction, and inflammation levels) between the groups treated with bisphosphonate (zoledronic acid) or PTH (teriparatide) combined with bone grafts and the control groups—was partially rejected. Significant differences were found in remaining bone graft, foreign body reaction, and inflammation levels across groups and time intervals, but not in new bone formation. The findings of our study in rabbit models showed that those receiving teriparatide had the highest new bone formation, as well as the lowest remaining bone graft, foreign body reaction, and inflammation levels in the 8th week compared to other groups. However, the teriparatide group exhibited the lowest percent of new bone formation in the 16th week. There were significant differences between the groups regarding remaining bone graft and foreign body reactions in both time intervals, in addition to inflammation levels in the 16th week.

 Consistent with our findings, a study by Belfrage et al.^22^ on rat models found that the amount of new bone formation in grafts soaked in zoledronic acid for 10 minutes was significantly less than that in the control group (*P* = 0.007), concluding that local administration of zoledronic acid enhances its inhibitory effect on new bone formation. In another animal study on rats, alendronate inhibited new bone formation compared to the control group, and similar to our study, the difference in the amount of bone formations between the alendronate and control groups was not significant.^23^ Yu et al.^17^ also showed the adverse effects of zoledronic acid on osteointegration. Another study on rabbits reported no significant differences in new bone formation and graft resorption between the alendronate and control groups.^24^ On the other hand, there is some evidence indicating higher osteogenesis, bone volume, and integration in animal models receiving bisphosphonates.^25,26^ The differences in the findings may be explained by variations in the type of animal models, the defect size, the sample size, the amount and concentration of bisphosphonates, the specific type of bisphosphonate (e.g., zoledronic acid or alendronic acid), the method of administration (i.e., local or systemic) and the timing of administration (i.e., immediate or delayed), as well as the timing of histopathological evaluation and the type of bone graft. These factors could contribute to contradictions by altering drug bioavailability, bone remodeling dynamics, or immune responses. For instance, systemic vs. local administration may lead to varying concentrations at the defect site, while species differences in bone metabolism (e.g., faster turnover in rats) could increase or mask the effects observed in rabbits.

 We found that new bone formation in the teriparatide group was 2.53 and 1.87 times higher than those in the positive and negative control groups at the 8th week, respectively. Similarly, Daugaard et al.^27^ demonstrated that the use of PTH in conjunction with bone grafting in canine models resulted in a 1.4-fold increase in new bone formation after four weeks. In this regard, PTH stimulates the proliferation and differentiation of osteogenic cells, increases the number of osteoblasts, enhances the synthesis of type 1 collagen, and promotes mineral deposition.^28^ In contrast, another study on healthy rabbits revealed that intermittent administration of PTH did not stimulate new bone area over a four-week period compared with the control saline group.^29^ The differences might be attributed to different follow-up periods. Such contradictions may arise from factors like PTH dosing frequency (intermittent vs. continuous), which influences anabolic vs. catabolic balance, or variations in defect severity, where milder defects in some studies might not require PTH’s stimulatory effects as much. Accordingly, new bone formation in the teriparatide group was lower than that in the other groups at week 16, which might be due to the predominance of osteoclastic over osteoblastic activities. It has been shown that during the first three months of using PTH, bone formation surpasses resorption. Then, a balance between new bone formation and bone resorption is established, and in the long term, bone resorption predominates over bone formation.^27^ Furthermore, the ratio of receptor activator of nuclear factor kappa-β ligand to osteoprotegerin determines the response to PTH, so that long-term exposures increase this ratio, leading to catabolic effects.^30^

 In the present study, the animals receiving zoledronic acid had higher percentages of remaining bone grafts at both time intervals. This might be attributed to the anti-osteoclastic activity of this compound as it has been shown that bisphosphonates induce apoptosis in osteoclasts.^31^ Other mechanisms include the reductions in osteoclast recruitment to the bone surface, osteoclast activity, and the rate of mineral content dissolution.^32^ In this regard, Belfrage et al.^22^ demonstrated that all the groups of rats receiving zoledronic acid had a higher amount of remaining bone compared to the control group. Another study in rats showed that alendronate reduces the number and size of bone resorption areas over 12 weeks.^33^ On the other hand, no significant differences were found in the amount of bone resorption between the control and alendronate groups in another study on rabbits.^24^ The conflicting findings might be due to inadequate control over study conditions and the effects of confounding factors or biases. For example, differences in animal age or baseline bone health could bias resorption rates, while unstandardized evaluation methods (e.g., manual vs. software-based histomorphometry) might introduce measurement variability, leading to contradictory outcomes. It has been determined that the breakdown of the extracellular matrix during the remodeling of bone graft releases bone morphogenetic protein (BMP)-7, promoting new bone formation. However, bisphosphonates inhibit the formation of BMPs, thereby preventing new bone formation.^34^ The teriparatide group showed lower remaining bone grafts in both 8th and 16th weeks, consistent with prior research.^35^ Özer et al.^18^ found that by the fourth week, the quantity of remaining bone graft was lower in the group receiving PTH compared to the control group, whereas by the 8th week, the control group showed higher levels. Discrepancies in results may arise from variations in the sample size, dosage, and the timing of histological evaluations.

 Two common and related processes that occur during grafting include inflammation and foreign body reaction. The present study showed significant differences in the frequency of foreign body reactions at both intervals, while only the 16th week showed significant inter-group differences in inflammation levels. The higher incidence of inflammatory responses and foreign body reactions observed in the bisphosphonate group in this study appears to be due to the greater percentage of remaining bone graft in this group, particularly at the 16th week.^36^ On the one hand, the study by Cheng et al.^25^ showed that the use of bisphosphonates immediately after bone grafting induces acute inflammation. On the other hand, other studies have indicated that bisphosphonates have anti-inflammatory and anti-neoplastic properties as it was shown that bisphosphonates do not cause any foreign body reaction during the repair of calvarial bone defects in rabbits.^37^ These opposing views may result from factors such as bisphosphonate type (nitrogen-containing vs. non-nitrogen-containing), which affects inflammatory pathways differently, or study-specific graft materials that interact variably with the drug. In the teriparatide group, the amount of residual bone powder in the 8th week was less than that in the positive control group due to the initial stimulation of osteoclastic activity on the surface of the graft material. Consequently, the occurrence of foreign body reactions and inflammatory responses in the 8th week was lower in this group compared to the other groups.

 The present study has some limitations. First, there was a limited sample size. Therefore, the results should be interpretated by considering this limitation. Both statistical and clinical significances should be noted since a small difference between groups may be statistically significant due to a large sample size but may not be clinically important. Conversely, a substantial difference that is clinically significant might not reach statistical significance due to an insufficient sample size.^38^ This small sample size may have reduced statistical power, potentially leading to underestimation of teriparatide’s early anabolic effects or bisphosphonates’ long-term inflammatory impacts, as non-significant trends in new bone formation could be false negatives. Second, rabbits have a different bone composition and a significantly higher bone turnover rate compared to humans. This should be taken into account when translating these results to human subjects. The faster metabolism in rabbits might exaggerate short-term effects of teriparatide (e.g., rapid shift to catabolic phase by week 16) and minimize bisphosphonates’ cumulative toxicity, limiting direct applicability to slower human regeneration processes. To control confounding variables more rigorously in future studies, we recommend larger sample sizes with power calculations to detect subtle effects, stratified randomization by factors like sex, age, and weight, inclusion of multivariate statistical adjustments for potential confounders (e.g., baseline bone density or hormonal status), standardized protocols for drug dosing and administration timing across studies, and incorporation of biomarkers to monitor bone turnover dynamically. Additionally, using multiple animal models or progressing to larger mammals could improve translational validity.

## Conclusion

 In summary, teriparatide and bisphosphonate exhibited opposing but potentially complementary effects on bone healing. Teriparatide improved new bone formation and resorption of the bone graft material in the short term, but in long-term, it diminished new bone formation. Additionally, teriparatide was associated with a lower foreign body reaction in the short term. In contrast, bisphosphonates reduced the resorption rate of the bone graft and new bone formation, while inducing a foreign body reaction. Overall, teriparatide showed more favorable outcomes. Future larger scale animal studies should investigate the combined use of teriparatide and bisphosphonates with optimized dosing.

## Competing Interests

 The authors declare that they have no conflict of interests.

## Ethical Approval

 The current study was approved by the Ethics Committee of Hamadan University of Medical Sciences, Hamadan, Iran (ethics code: IR.UMSHA.REC.1399.110). This study was carried out in strict accordance with the recommendations in the Guide for the Care and Use of Laboratory Animals of the National Institutes of Health. All efforts were made to minimize suffering.
